# Genomic Data Reveal a Complex Making of Humans

**DOI:** 10.1371/journal.pgen.1002837

**Published:** 2012-07-19

**Authors:** Isabel Alves, Anna Šrámková Hanulová, Matthieu Foll, Laurent Excoffier

**Affiliations:** 1 CMPG, Institute of Ecology and Evolution, Berne, Switzerland; 2 Swiss Institute of Bioinformatics, Lausanne, Switzerland; 3 Population and Conservation Genetics Group, Instituto Gulbenkian de Ciência, Oeiras, Portugal; Aarhus University, Denmark

## Abstract

In the last few years, two paradigms underlying human evolution have crumbled. Modern humans have not totally replaced previous hominins without any admixture, and the expected signatures of adaptations to new environments are surprisingly lacking at the genomic level. Here we review current evidence about archaic admixture and lack of strong selective sweeps in humans. We underline the need to properly model differential admixture in various populations to correctly reconstruct past demography. We also stress the importance of taking into account the spatial dimension of human evolution, which proceeded by a series of range expansions that could have promoted both the introgression of archaic genes and background selection.

## Introduction

Until recently, the out-of-Africa model of human evolution was favoured by most genetic analyses, but this model collapsed when the sequencing of the Neanderthal genome revealed that 1%–3% of the genome of Eurasians was of Neanderthal origin. At the same time, refined analyses of modern human genomic data [1]–[3] have changed our view of evolutionary forces acting on our genome. While most people assumed that the out-of-Africa expansion had been characterized by a series of adaptations to new environments [4]–[6] leading to recurrent selective sweeps [7], our genome actually contains little trace of recent complete sweeps [2], [3], [8] and the genetic differentiation of human population has been very progressive over time, probably without major adaptive episodes [9]. In this review, we detail these changes of paradigm and we discuss their implication for future studies of human diversity.

## Interbreeding between Modern and Archaic Humans

**Figure 1:**
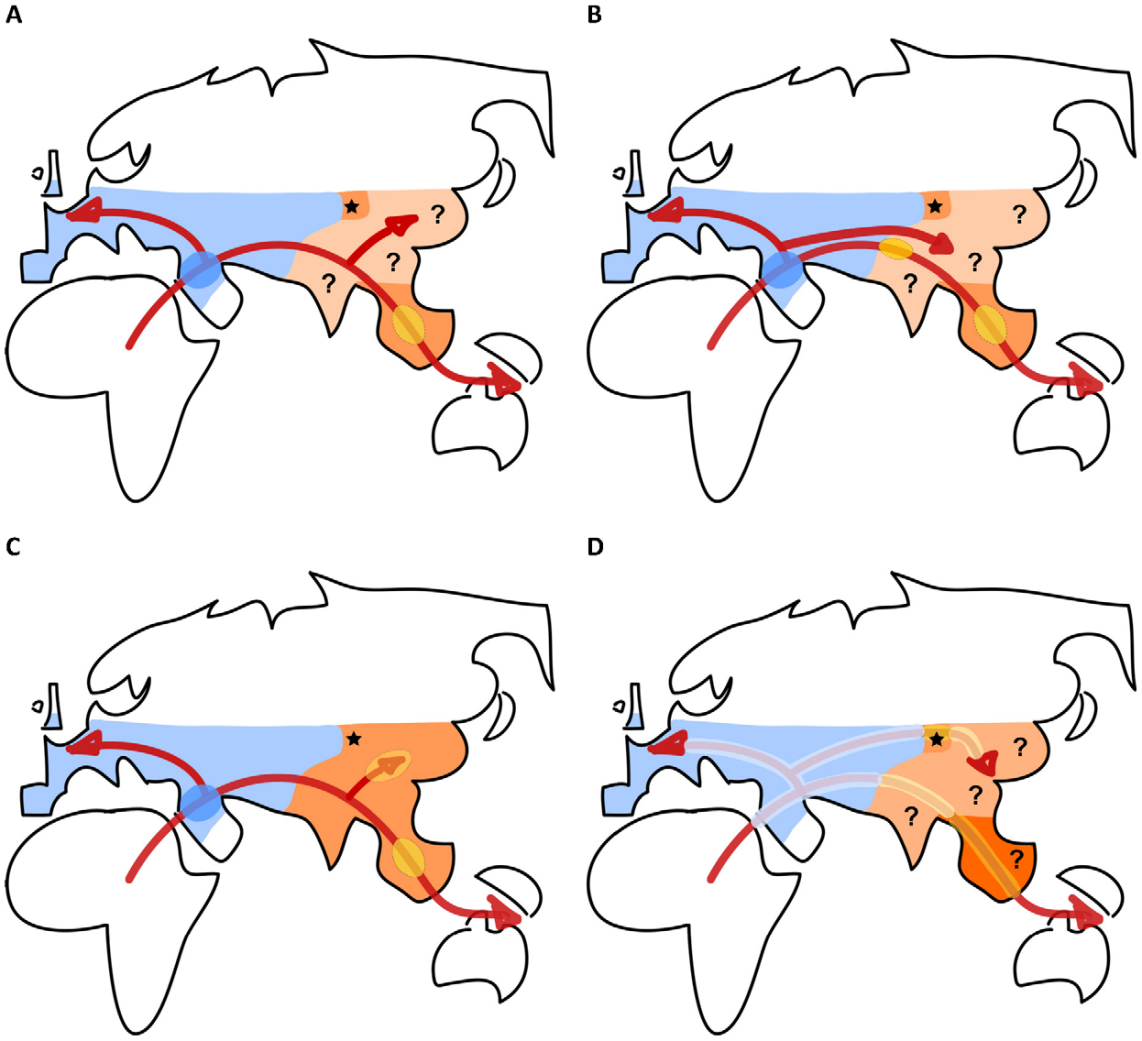
Sketches of different scenarios of human dispersal and admixture with archaic human populations during their range expansion out of Africa. Red arrows indicate approximate migration routes. Neanderthal range is in blue, Denisovan range(s) in orange, and the location of the Denisova site is indicated as a black star. Question marks in the Denisovan range indicate uncertainty on Denisovan hominin presence. Filled ellipses indicate potential places of admixture in scenarios (A–C). (A) Scenario of Reich et al. [15], [16] with pulses of admixture between modern humans and Neanderthals (dark blue ellipse) and between modern humans and Denisovans (yellow ellipse). (B) Scenario of Rasmussen et al. [24] with two waves into Asia. Denisovan admixture in Oceanians would have occurred during the first wave, possibly at different places during the migration. (C) Scenario of Skoglund and Jakobsson [17], with distinct Denisovan admixture events in Oceanians and East Asians. (D) Extension of the spatially explicit scenario of Currat and Excoffier [25] postulating a continuous admixture between modern humans and archaic hominins along migration routes overlapping with archaic hominin ranges. Different shades of orange indicate potentially different archaic hominin populations in Asia.

In line with previous studies [10]–[12] which suggested that some aspects of human genomic diversity were incompatible with a complete replacement of archaic hominins, evidence for admixture between humans and Neanderthals emerged from the first analysis of a complete Neanderthal genome [13]. Indeed, the presence of a significant excess of Neanderthal-derived alleles in Eurasian populations as compared to Africans has been interpreted as resulting from an admixture episode between the ancestors of Eurasians and Neanderthals somewhere in the Middle East [13] (Figure 1A). Even though the existence of a very ancient population subdivision in Africa from which both Neanderthals and Eurasians would have emerged could lead to similar patterns [14], the maintenance of such a subdivision over tens of thousands of generations seems unlikely. The sequencing of another archaic hominin from the Denisova cave in the Altaï mountains in Siberia has further revealed that Papua New Guineans showed signs of introgression from this archaic human [15]. Further studies of 33 populations from Southeast Asia and Oceania [16] showed that Denisovan admixture was actually present in other Oceanians, Melanesians, Polynesians, and east Indonesians but was virtually absent in mainland east Asians (but see [17] for evidence of possible Denisovan introgression on the Asian continent). Overall, these genomic analyses of admixture suggest that 1%–3% of the genome of all Eurasians and native Amerindians is of Neanderthal origin [15], and that Papua New Guineans and Australians have another 3.5% of their genome of Denisovan origin [16]. The out-of-Africa model of human evolution, which posited a complete replacement of archaic by modern humans in Eurasia, thus needs to be modified to include a limited assimilation of archaic genes, but the fact that most of the genetic variation observed in extant non-African populations comes from Africa remains true.

The finding of archaic admixture in Eurasia gives credit to previous statistical analyses, which have suggested the presence of archaic material in Eurasian and African populations [11]. In order to better assess the possibility of admixture in Africa, Hammer and colleagues [18] recently looked for signals of archaic admixture in two African hunter-gatherer populations and in a West African farmer population using a set of 61 non-coding autosomal loci. They found that an absence of admixture could not explain observed patterns of linkage disequilibrium in the hunter-gatherer populations, suggesting that they were potentially admixed with a yet unknown archaic hominin. A model including admixture suggested a recent admixture event (10–40 Kya) with a very divergent archaic population. While the confidence intervals of the archaic admixture rate are extremely broad (ranging from 0% to 100%), point estimates suggest that admixture was low and limited to 0.5%–2%. It remains to be shown if this estimate would be sensitive to other forms of admixture (e.g., with Bantu recent input into Pygmies and San [19]–[21]).

### Where and How Did Admixture Occur?

There is thus both direct [13], [15] and indirect [11], [18] evidence for archaic admixture on four continents, suggesting that modern humans have not been totally genetically isolated since their emergence, some 150–200 Kya in East Africa [22], [23]. However, there is still quite some discussion about the place, the timing, the exact numbers of admixture events, and the biological implications of these interbreeding events (see Figure 1). The finding of almost equal levels of Neanderthal introgression in all Eurasians has been interpreted as evidence for a unique pulse of admixture in the Middle East between Neanderthals and the ancestors of Eurasians [13] (Figure 1A). The fact that Denisovan admixture had been first evidenced in Papua New Guineans suggested that admixture had occurred as a single pulse in Southeast Asia, after the separation of the ancestors of Oceanians and other Asians [15], [16] (Figure 1A). The analysis of an Australian genome has confirmed the presence of Denisovan admixture in Australians [24] and suggested that admixture occurred during a first early wave of colonization towards Oceania, either in Southeast Asia or earlier in Eurasia (Figure 1B). A reanalysis of a large human SNP database and its comparison with Denisovan-derived alleles has suggested the presence of Denisovan admixture in East Asians, albeit at lower levels than in Oceanians [17], which could have occurred at a different place than for Oceanians, somewhere in East Asia (Figure 1C). Contrastingly, Currat and Excoffier [25] introduced a spatially explicit model of interbreeding between Neanderthals and Eurasians that could occur over the whole Neanderthal range (Figure 1D). They obtained similarly low levels (1%–3%) of Neanderthal introgression in both Europe and China if interspecific exchanges were locally extremely limited (only 200–400 interbreeding events over the >6,000 years of co-existence between the two species). An extension of this scenario to Denisovan admixture would imply that modern humans could have hybridized along all migration routes overlapping with the range(s) of archaic humans (Figure 1D). The fact that the largest levels of Denisovan introgression are found in Oceanians raises the question of a potential discontinuity in the Denisovan range (Figure 1A, 1B) or of a genetic differentiation of archaic hominins living in different ecosystems (Figure 1D). Alternatively, modern humans could have admixed with other hominins [26], and/or inferred hominin introgression could result from the sharing of some derived sites between Neanderthals, Denisovans, and unidentified archaic hominins. A scenario involving an unsampled Eurasian archaic hominin has received support from a recent study [27] showing the presence of a highly divergent (>3 Mya) haplotype of the innate immune gene *OAS1*. This deep lineage is found at high frequencies in Oceania (and at lower frequencies up to Pakistan). This DNA segment is more closely related (0.6 Mya divergence) to the Denisova sequence than to the Neanderthal sequence, which is itself closer to the human reference sequence. It has been speculated [27] that this fragment had introgressed from a more archaic hominin than Denisovans, who could have been themselves introgressed earlier.

### Genomic Distribution of Archaic Admixture Is Still Lacking

Our understanding of the exact sequence and location of admixture events would highly benefit from a more precise knowledge of the nature and the distribution of Neanderthal segments in our genome. Unfortunately, current estimations of introgression levels are based on a statistic measuring a genome-wide difference in the proportion of archaic-derived alleles between two human populations [13], [14], so that the genomic distribution of introgressed segments is still unknown. However, in addition to the *OAS1* segment mentioned above [27], several authors have recently argued they had identified candidate regions harboring archaic haplotypes [13], [28], [29]. These regions usually show highly divergent haplotypes with very little evidence for recombination [30]. A dozen genomic regions where Eurasians have haplotypes much more divergent than Africans and a high proportion of derived Neanderthal alleles have been proposed as candidates for Neanderthal introgression [13]. More recently, an X-linked haplotype (B006) in an intron of the dystrophin (*dys44*) gene, almost absent from Africa but with 9% average frequency outside Africa, has been proposed to be of Neanderthal origin [29]. It is close to the ancestral X haplotype, shares 2/3 of derived alleles with Neanderthals, and has little associated diversity, suggesting a recent origin in humans. Another study has also suggested that several immune-related HLA class I alleles in humans could be of Denisovan origin and that they helped Eurasian populations build their immunity [28]. Whereas the hypothesis of an adaptive introgression is highly seductive, its support is relatively thin. “Denisovan” HLA class I alleles are currently not confined to Oceania but are found widespread in Asia. Moreover, the strongest argued case of Denisovan allelic ancestry (HLA-B*73) is actually not found at all in the Denisovan genome and is presently distributed in western Asia, well in the former Neanderthal range. One should therefore be extremely cautious not to assume that each very divergent haplotype found in humans is necessarily of archaic origin, as cases of incomplete lineage sorting are not rare between higher primates [31], especially in the HLA system where trans-specific polymorphism is facilitated by balancing selection [32]. However, if some introgressed genes were really advantageous, they should have spread and fixed in the human population, but as discussed below there is no widespread signature of strong selective sweeps in Eurasia.

It may nevertheless be valuable to identify further genomic regions of potential archaic origin. Previous candidate regions have been identified, as they showed a much larger time to the most recent common ancestor (TMRCA) in Eurasia than in Africa. This signal may, however, not be optimal, since if Neanderthals and modern humans diverged only 270–440 Kya [13], the presence of some Neanderthal lineages in a Eurasian population should not greatly affect the TMRCA unless Eurasian ancestors had gone through a very drastic bottleneck, which does not seem the case [33]. Indeed, modern human segments show a TMRCA modal value around 1.5 Mya [34], well beyond the divergence with Neanderthals/Denisovans. Assuming that large TMRCA is a true signal of admixture, one would expect to see many more regions of potential archaic origin in Oceanians, which show higher levels of archaic introgression than mainland Eurasians (5% versus 1.5%, respectively, [16]). Until the diversity of archaic haplotypes along the chromosomes is better assessed, other signals of introgression might be more discriminant to find archaic segments in our genomes, like spikes of positive Tajima's D or measures of tree imbalance [35].

### Can We Still Analyse Human Genetic Data without Taking Admixture into Account?

**Figure 2:**
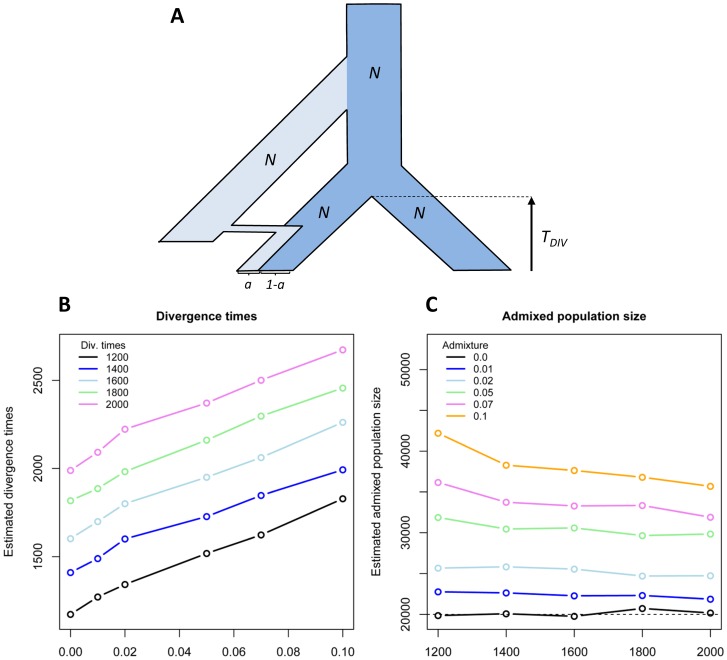
Biased estimation of divergence time and population sizes in case of admixture. (A) Model of population divergence and admixture: one of two populations having diverged *T_Div_* generations ago has received a fraction *a* of its genes from another unsampled population that diverged 14,000 generations ago (350,000 y assuming a generation time of 25 y). All populations sizes are assumed to consist of *N* = 20,000 haploids. (B) Estimated divergence time as a function of initial admixture rate *a*. (C) Estimated admixed population size for different divergence times and admixture rates. Simulated data consisted of 400,000 segments of 50 bp, thus totalling a 20-Mb DNA sequence. Parameters are estimated by maximizing the probability of the observed joint site frequency spectrum (SFS) [68], where the expected SFS is estimated by simulation following the approach of Nielsen et al. [69].

If human populations do not all have the same level of archaic introgression, the current genetic structure of human populations might be partly shaped by differential admixture. Estimates of population sizes and divergence times between human populations should thus be affected by past admixture events. The divergence time between an admixed and a non-admixed population should be overestimated if admixture is not properly modelled. Similarly, the effective size of admixed populations should be overestimated as archaic lineages inflate genetic diversity. In Figure 2, we report a simulation study of this bias in a very simple case of population divergence without migration. The overestimations of divergence time and admixed population size are almost linearly increasing with admixture rate (Figure 2). For instance, a divergence time of 1,600 generations (40,000 y assuming a 25-y generation time) is perfectly recovered if none of the populations is admixed, but is overestimated by 100 generations (2,500 y) with 1% admixture in one population, and already by 350 generations (8,750 y) with 5% admixture. Even though our simulated scenario is unrealistically simple, it is likely that differential admixture should affect population genetic affinities under more complex models of population differentiation. The proper interpretation of human genetic affinities should thus probably be re-evaluated in the light of these results. In particular, the divergence between Africans and Oceanians (showing up to 5% archaic admixture [16]) could be more recent than previously reported (62–75 Kya [24]). It remains unclear whether the method used by Rasmussen et al. [24] to date this divergence is also sensitive to differential introgression, but, if that was the case, the colonization wave to Oceania thought to well predate that towards East Asia [24] could have occurred at roughly the same time once differential admixture had been taken into account.

## Missing Signals of Adaptation in Our Genome

Most methods aiming at detecting recent episodes of selection in humans have been designed under the paradigm that adaptations were mainly driven by classical positive selection: beneficial alleles should go to fixation, strongly reducing diversity and increasing levels of linkage disequilibrium in the surrounding regions. Such selective sweeps would thus strongly affect various aspects of molecular diversity within and between populations (e.g., [36]). Several lines of evidence support the past action of positive selection, such as increased levels of population differentiation in or close to genic regions [3], [37], increased diversity with distance from coding regions [38], or lower diversity and increased population differentiation in regions of low recombination where selective sweep should be more efficient [8], [39]–[41]. However, this paradigm has been recently eroded as it has been realized that our genome does not show many sites that are fixed between human populations [2], [38], and that fixed differences are always between populations from different continents [3], suggesting that strong adaptive events rarely occurred in response to local adaptation.

### Background Selection Can Explain Most Observed Patterns of Polymorphism

**Figure 3:**
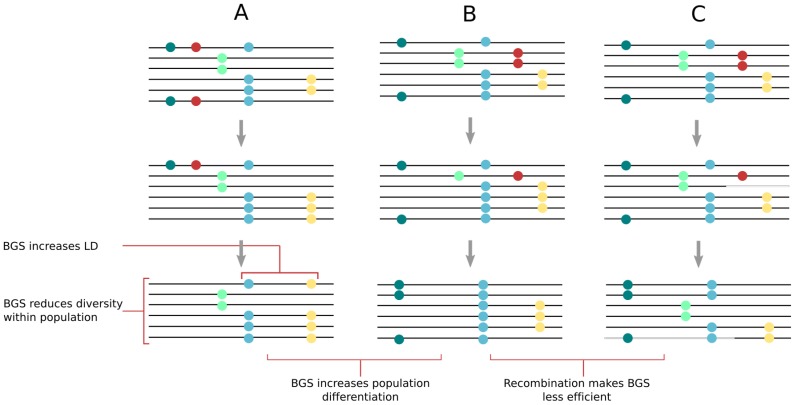
Effect of background selection (BGS) on molecular diversity within and between populations. After a BGS episode, deleterious mutations (shown in red) are eliminated together with neutral mutations on the same chromosome, leading to reduced diversity. For illustrative purposes, initial neutral diversity is identical in all cases (A–C). Comparison of cases (A) and (B) shows that different BGS episodes will contribute to populations' genetic differentiation. Comparison of cases (B) and (C) shows that recombination reduces the effect of BGS, maintaining diversity, and reducing linkage disequilibrium (LD) as well as population differentiation (compare final states in [A] and [C]).

Three recent observations have further shaken the paradigm of positive selection. First, it has been realized that regions showing high levels of differentiation between continents (high *F_ST_*) were not associated with large levels of linkage disequilibrium, suggesting that allele frequency shifts occurred long ago and not because of recent adaptive events [3], [9]. Second, it was shown that the reduction in diversity is practically identical around non-synonymous or synonymous sites [2], suggesting that the diversity trough in genic regions is not due to positive selection acting on amino-acid changing mutations, but better fits a model of background selection, which eliminates strongly deleterious mutations in functional regions (see e.g., [42], [43] for recent reviews on background selection). Finally, models with selective sweeps have been shown to lead to an overly strong negative correlation between levels of synonymous polymorphism and non-synonymous divergence [8], whereas models of background selection fit the observed correlation. Evidence is thus building that background selection can explain most aspects of observed patterns of polymorphism. As illustrated in Figure 3, background selection lowers levels of diversity at linked sites [44], increases levels of both linkage disequilibrium [45] and population differentiation [46], and has an effect similar to a reduction of the effective population size [47], which locally lowers coalescence times [48] but also distorts the site frequency spectrum, which shows an excess of rare variants [45]. The effects of background selection on associated diversity should also be more pronounced in regions of low recombination [42] and thus provide an alternative explanation for the positive correlation between recombination rates and levels of diversity [44]. Because background selection can explain most aspects of human genetic diversity, it does not mean that adaptive events driven by positive selection have not occurred in recent or past human evolution (e.g., [49]), but they might not be that widespread and detecting their signal might be more difficult than anticipated. However, while we emphasize here the potentially important role of background selection, it is clear that other forms of selection (see e.g., [9], [50]) or other purely demographic factors (e.g., [3], [51], [52]) have certainly played an important role in shaping human genetic diversity.

### Alternative Explanation for a Lack of Complete Sweeps

At a single locus, selection on standing variation [53] as well as recurrent mutation or migration [54] can result in soft sweeps where a given beneficial mutation is fixed on different chromosomal backgrounds. Positive selection acting simultaneously on several alleles [55] or sequentially over time on different alleles can lead to incomplete sweeps, where beneficial mutations are not necessarily fixed. However, most phenotypic traits are controlled by several loci, so that Pritchard and colleagues [9], [50] have argued that an absence of hard sweeps in humans could be due to polygenic adaptation from standing variation. This model assumes that most traits are controlled by multiple genes and that an adaptive event will result in the simultaneous increase in frequency of different alleles at multiple unlinked loci. After a selective event shifting the phenotype distribution around a new optimum, several selected alleles would have increased in frequency without any one being necessarily fixed.

## Necessity and Benefits of Spatial Scenarios of Human Evolution

A proper scenario of human evolution should explain both the current distribution of archaic introgression given the past distribution of archaic hominins and the likely migration routes of modern humans. Spatially explicit methods simultaneously modeling range expansions and interbreeding use observed levels of admixture to assess migration and demographic processes, and thus bring additional information on the biology of our species. Whereas the surfing of neutral polymorphism during range expansions has been shown to lead to molecular signatures similar to selective sweeps [52], [56], the spread of deleterious alleles during range expansions could make background selection more potent. Spatially explicit scenarios of evolution can thus make better use of available information and provide new explanations for observed molecular diversity patterns.

### Implications of Spatial Models of Admixture

Scenarios of pulses of admixture do not provide any explanation for why interbreeding only occurred in some places and why archaic hominins disappeared in regions where no admixture took place. Contrastingly, scenarios of continuous admixture during range expansion explicitly posit that archaic hominins disappeared due to their interaction or competition [57], [58] with the first human invaders. This is not very flattering for our species, but it provides a hypothesis framework that could be tested with archaeological and future genomic data. Moreover, a spatially explicit model of admixture has provided information on the frequency of interbreeding events [25], and it predicts an asymmetric introgression from archaic to modern humans [13], even if archaic populations have been much less numerous than invading modern humans [59]. High levels of introgression from the local population are indeed expected if on average more than one gene introgresses the newly invading population at any given location on the wave front [60], [61]. Had this happened, modern humans would have become archaic and the expansion would have stopped. Note also that the large levels of introgression expected after a range expansion with interbreeding argue against a complete replacement of the European Palaeolithic people by Neolithic populations expanding from the Middle East [62]. It implies that the presence of any European-specific component of Neanderthal admixture should not have been totally erased by later Neolithic expansions in Europe. A Palaeolithic introgression signal should thus be still visible in Europe, allowing one to distinguish between hypotheses of single pulses of admixture (Figure 1A; [13]) and of continuous admixtures with different archaic populations (Figure 1D).

### Colonization Routes through Eurasia Mapped by Admixture?

The patterns and levels of archaic admixture in current Eurasians should be informative about modern humans' migration routes in Eurasia if they had hybridized with genetically distinct archaic populations or species. For instance, Europeans and Asians could show distinct components of Neanderthal admixture if they had admixed with European and central Asian Neanderthals [25], respectively. A detailed inventory of the genomic diversity of archaic hominins should not only allow us to better define their past range, but also make it possible to geographically map the most likely places of past admixture events, test the hypothesis of pulses of admixture, and reconstruct the migration trajectories of the ancestors of human populations from different continents. Additional statistical analyses of extant data could also allow us to date past admixture events (e.g., [63]), which could help us distinguish between scenarios of ancient admixture pulses in the Middle East and more recent interbreedings in peripheral regions.

### Spatial Expansions Can Promote Background Selection

Taking into account the fact that human populations went through recurrent range expansions could also help us understand the prevalence of background selection. It has indeed been shown that in addition to beneficial and neutral mutations, deleterious mutations could surf during range expansions and thus temporarily increase in frequency at the wave front [64], [65]. This spread of deleterious alleles during spatial expansions is made possible by low population densities on wave fronts and a high growth rate favoured by a relaxation of competition for resources [66], which increases the role of drift and limits that of selection. Deleterious mutations can thus behave as neutral mutations and accumulate on expanding wave fronts. Once population densities increase in the range core, selection can become stronger than drift: purifying and background selection can progressively operate. If confirmed, this phenomenon could explain the observation in European populations of an excess of slightly deleterious alleles [67], which could have accumulated during Palaeolithic and Neolithic range expansions, but more work is needed to fully understand the interaction of beneficial and deleterious mutations in expanding populations.

## Conclusions

As James F. Crow would have put it, in human evolution the questions have remained the same but the answers have changed. Genomics has revealed that the genome of Eurasians is partly of archaic origin, and genome-wide patterns of diversity have not revealed expected signals of adaptive selection in humans. The sequencing of additional archaic hominins should be helpful to distinguish between alternative scenarios of admixture, infer the timing and the geographic location of admixture events, and assess human migration routes over Eurasia. Archaic admixture can also seriously impact estimated human demography, which should be revisited to account for differential introgression among human populations. Scenarios of human evolution need to be geographically coherent and integrate range expansions during which deleterious mutations can readily surf and accumulate on wave fronts, giving later fuel to background selection. Whereas our view of human evolution has drastically changed over the past few years, it would be pretentious to believe we now know the true history of modern humans and that we have identified all selective forces that have shaped the diversity of our genome. However, progress in the analysis of modern and ancient genomes is likely to soon provide the data that will allow us to test complex scenarios of human evolution and contrast the role of various selective forces that are currently or were acting in our genome.
